# Environmental enrichment in commercial flocks of aviary housed laying hens: relationship with plumage condition and fearfulness

**DOI:** 10.1016/j.psj.2022.101754

**Published:** 2022-01-30

**Authors:** Fernanda M. Tahamtani, Kathe Kittelsen, Guro Vasdal

**Affiliations:** Animalia, Norwegian Meat and Poultry Research Centre, 0513 Oslo, Norway

**Keywords:** laying hen, environmental enrichment, feather pecking, fearfulness

## Abstract

Management strategies can have positive effects on laying hen welfare, including prevention of damaging behavior, aggression, and fear, particularly by using environmental enrichment (**EE**). However, few studies have investigated the effects of the provision of EE in commercial aviary flocks. This knowledge gap is particularly significant considering the increasing numbers of non-beak trimmed hens worldwide kept in aviaries. The aim of this study was to survey and investigate the relationship between commercially applied EE and plumage condition and fearfulness in Norwegian flocks of loose-housed laying hens. Forty-five indoor multi-tiered aviary-system flocks of laying hens from across Norway were visited at the end of lay (range: 70–76 wk of age). The flocks consisted of either Lohmann LSL (n = 30) or Dekalb White (n = 15) non–beak-trimmed hens. During the visit, the researchers collected data on the farmers’ use of the following five types of enrichment: pecking stones, gravel, oyster shells, grains scattered in the litter, and “toys”. Feather loss was assessed individually in approximately 50 hens per flock and scores were awarded using a 3-point scale (0–2) for each of the following body parts: head, back/wings, breast, and tail. Finally, a novel object test was performed in 4 different locations in each house. The results showed that damage to the tail feathers was correlated positively to the first age of provision of toys (Pearson correlation coefficient = 0.41; *P* = 0.051) and negatively to the amounts of gravel stones provided (Pearson correlation coefficient = −0.43, *P* = 0.02). No other associations between the welfare indicators and the provision of EE objects were found, likely because of the low variation of enrichment provision. The present study showed that the provision of EE objects such as toys and gravel stones can have significant benefits to the condition of laying hen plumage. This study also adds to the body of literature supporting the importance of early life experiences on the behavioral development of laying hens and on their welfare at older ages.

## INTRODUCTION

Consumer concern for the welfare of animals, particularly farm animals, has been increasing during the past several decades. Researchers and consumers alike recognize that good animal welfare depends not only on the absence of negative components, such as pain, disease, injury, hunger, and boredom, but also on the active promotion of a positive subjective experience. The animals must feel good, meet their behavioral needs, and have a life worth living (Dawkins, 2004; Webster et al., 2004). For this reason, non-cage housing systems for laying hens, such as multi-tiered aviaries, are increasingly used in commercial egg production in the last decade, as these systems allow for greater possibility of movement and expression of natural behaviors (Schuck-Paim et al., 2021). However, they may still pose welfare concerns. Existing literature reveals that some of the major welfare challenges described for layers housed in the loose-housing systems are damaging behaviors such as feather pecking and cannibalism (Heerkens et al., 2015), aggression, and fear, sometimes resulting in panic-induced smothering (Bright and Johnson, 2011). Management strategies can have positive effects on laying hen welfare, including prevention of damaging behavior, aggression, and fear, particularly by using environmental enrichment (**EE**). EE is defined as an improvement of the environment of captive animals that increases the behavioral opportunities of the animal and leads to an enhancement of its biological function (Newberry, 1995). The provision of hay bales, for example, can reduce the incidence of gentle feather pecking, and increase perching and dust bathing behavior (Daigle et al., 2014).

Feather pecking is largely accepted as redirected ground pecking due to an unsatisfied behavioral need for either foraging (Hoffmeyer, 1969; Blokhuis and Arkes, 1984) or dustbathing (Vestergaard and Lisborg, 1993; Vestergaard et al., 1993). The provision of litter can reduce the incidence of this behavior as well as improve plumage condition and reduce mortality of adult hens (Blokhuis and van der Haar, 1989; Green et al., 2000; Bestman et al., 2009; Tahamtani et al., 2016). Furthermore, the provision of EE can reduce the incidence of feather pecking, aggressive pecking and improve plumage condition during both the rearing and laying period (Huber-Eicher and Wechsler, 1998; Jones and Carmichael, 1998; Jones et al., 2002; McAdie et al., 2005; Tahamtani et al., 2016; Zepp et al., 2018).

Furthermore, access to roughage in the form of maize silage, barley-pea silage and carrots has been shown to reduce plumage damage, mortality, and the incidence of severe feather pecking (Steenfeldt and Nielsen, 2015; Riber et al., 2018). In most countries, there is no legal requirement for the provision of roughage to laying hens. However, as part of their management of beak-intact birds, some producers have introduced EE such as roughage or other kinds of occupational material, for example pecking stones, to prevent the development of damaging behavior. Indeed, limitation of litter during the rearing period can promote plumage damage and feather pecking during the production period (de Haas et al., 2014; Tahamtani et al., 2016). In addition, providing litter during the rearing period and EE objects during the production period have also been connected with reduced fearfulness in layer flocks (de Haas et al., 2014; Brantsæter et al., 2017).

Nevertheless, in order to be truly useful, EE must be a “functionally relevant” object, that is it must bring meaningful and positive change in animal behavior and welfare and be both practical and economically beneficial (Newberry, 1995; van de Weerd and Day, 2009). Furthermore, EE objects must be tested to ensure their purpose. For example, Lindberg and Nicol (1994) provided laying hens with an operant feeder, which increased the amount of pecking that had to be performed to receive a food reward, with the expectation that it would redirect pecking from feathers to the operant feeders. However, the use of these operant feeders was associated with higher rates of feather pecking compared to ad libitum feeding, likely due to increased frustration (Lindberg and Nicol, 1994). It is important, therefore, to assess the effect of commercially applied EE objects on laying hen welfare. The EE objects currently in use are considered practical and economically neutral by the farmers. However, few studies have investigated the effects of the provision of EE in commercial aviary flocks of laying hens. This knowledge gap is particularly significant considering the increasing numbers of non-beaktrimmed hens worldwide kept in aviaries.

The aim of this study was to survey and investigate the relationship between commercially applied EE and selected measures of behavior and welfare in Norwegian flocks of loose-housed laying hens. To this end, commercial flocks of laying hens were visited near the end of the production period and data was collected on the type, quantity, and frequency of EE provision. In addition, the birds were assessed for plumage condition and fear responses to a novel object. It was predicted that increased environmental complexity (i.e., EE provision from an earlier age, in higher quantity and in increased frequency) would lead to an improvement of plumage condition and a reduction in fearfulness.

## MATERIALS AND METHODS

### Animals and Housing

This study was conducted between May 2020 and June 2021 and included 45 indoor multi-tiered aviary-system flocks of laying hens from across Norway. The flocks consisted of either Lohmann LSL (n = 30) or Dekalb White (n = 15) non beak-trimmed hens and flock sizes ranged from 5,300 to 19,000 hens (mean = 7,921). The hens were housed under a 14-h light/ 10-h dark schedule and ad libitum access to both feed, via a chain dispersal system, and water via drinking nipples. The flocks were managed according to standardized practices with regards to feed, water, ventilation, litter, and lighting (KSL, 2020). Mean light intensity ranged from 1 to 8 lux between houses as measured with a luxometer (Extech LED meter LT40, FLIR Commercial Systems Inc., Nashua, NH) The pullets arrived at the farms at approximately 16 wk of age and were kept until 78 wk when they were depopulated following standard commercial practices for Norway. All farms had similar layout, with 3 tiers above the floor, feed, and water lines on tiers 1 and 2, nest boxes on tier 2, and perches on tier 3. The houses were about 12-m wide, with wood shavings litter covering a floor area ranging from 385 m^2^ to 1,000 m^2^ that extended around and under the tiered aviary structures.

### Farm Visits and Data Collection

The flocks (1 flock/farm) were visited once near the end of the production period, between 70 and 76 wk of age. Each flock was visited by one of three researchers, such that each researcher visited 15 flocks. During the visit, which lasted approximately 2 h, aspects of the environment in the hen room and the behavior and physical condition of the hens were recorded. All visits were conducted at approximately 09:00 h, during the light hours of the light cycle. Normal routines of the system (e.g., feeder chains, light intensity) were not altered during the assessment. For biosecurity reasons, only one flock was visited per day, and the visiting researcher used the appropriate disposable clothing (body suit, mask, and shoe covers).The percent mortality of the flock was recoded as reported by the farmer when these data were available.

#### Plumage Condition and Wound Score

Feather loss was assessed individually in approximately 50 hens per flock using the NorWel method (Vasdal et al., 2022). All researchers had previous experience and training in this method. The assessment was done by observing the birds without handling to minimize stress and disturbance of the flock. Choice of hen was based on the following principle: one hen was chosen and the hen second closest to that hen was visually scored. Only hens that had all assessed body parts visible to the observer were scored. The observer walked calmly along the corridors and scored hens from all parts of the house (floor, slats, ramps, perches, etc.). Scores were awarded using a 3-point scale (0–2) for each of the following body parts: head, back/wings, breast, and tail. A score 0 was given when there was no feather loss at that body part. Score 1 was given when feathers were missing from an area <5 cm in diameter in the body part. If the featherless area was >5 cm in diameter, that body part was given a score of 2. The condition of the tail feathers was not assessed in the first 3 flocks visited and, therefore, these data are only available from 42 flocks.

The dirtiness of the plumage was also scored on a 3-point scale from 0 to 2. A score 0 was given for clean feathers; score 1 was given when feathers had clear and dark staining covering at least 10% of the body part; and score 2 was given when feathers had clear and dark staining covering at least 50% of the body part. Finally, the presence of wounds (i.e., visible marks related to fresh or older wounds) was scored on a dichotomous scale for each of the following body parts: head, back and vent.

### Novel Object Test

**Figure 1:**
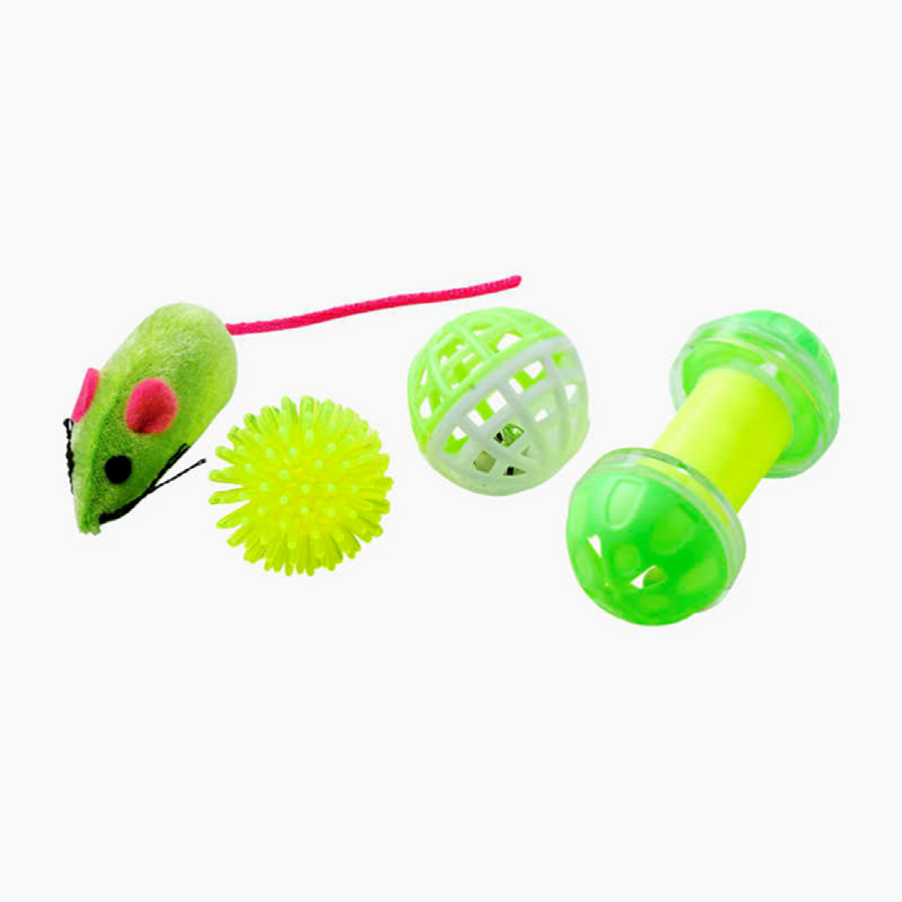
The objects used during the novel object test. At each of the four locations in which the test was conducted in each flock, one of these objects was randomly chosen for use until all four were used in the same flock (Katteleker, Biltema, Norway).

Fearfulness was assessed by a novel object (**NO**) test, as described in the Welfare Quality Assessment protocol for poultry (Welfare Quality, 2009) and previous protocols conducted on loose housed hens (de Haas et al., 2014; Brantsæter et al., 2017). To generate a representative average for the flock, the NO test was performed in four locations in the hen house. The tests were thus carried out in all corridors, and at different distances to the door. At each location, one of 4 NOs were randomly selected (Katteleker, Biltema, Norway; Figure 1). The NO was placed on the litter in the corridor and the researcher stepped slowly backward 10 steps. After placement, every 10 s, the researcher counted the number of hens within bird length (approx. 25 cm) of the NO and the number of pecks on the NO. The test lasted a total of 2 min.

### Environmental Enrichment

During the visit, the researchers made notes on the farmers’ use of the following 5 types of enrichment: pecking stones, gravel, oyster shells, grains scattered in the litter and “toys”. The most common type of toy used was cut-up pieces of manure belt. Other “toys” commonly observed were plastic balls, pieces of cardboard boxes and empty ice cream boxes. The producers were questioned about what types of enrichment they provide the hens, when they first provide each type of enrichment, the amount of each EE provided and how often they replenish the EE, where relevant. The frequency of replenishing of each EE was scored as either “daily” if every day, “weekly” if at least once a week, or “monthly” if at least once a month.

### Ethical Statement

Because the study did not involve any animal handling, experimental manipulations, or invasive procedures, it was exempt from approval of animal use by the Norwegian Food Safety Authority (Norwegian Regulations on Use of Animals in Research, 2015).

### Statistical Analyses

Statistical analyses were performed using the software SAS 9.4. The scores for feather loss in each of the 4 body parts (i.e., head, back/wings, breast, and tail) were averaged per flock. In addition, a total body score for feather loss was calculated as the sum of the 4 body parts assessed with a maximum score of 8 (i.e., the sum of four scores, each with a maximum of 2). The individual scores for wounds were also averaged per body part. The individual scores for plumage dirtiness were averaged per flock. The results from each NO test per flock (one for each type of NO) were summed and averaged to yield an average number of hens approaching the NO/flock. Plumage dirtiness and wounds scores were overall very low and were, therefore, not statistically analysed. Only 6 of the flocks visited (13.3%) provided less than 4 of the 5 types of enrichment surveyed. Therefore, it was not possible to analyze the number of EE types used. For the same reason, we could not analyse the effects of the presence or absence of the specific types of EE.

The relationships between the age at which each type of enrichment was first provided, the amount of enrichment provided and the scores for feather loss, flock mortality, and the NO test were assessed using Pearson correlations. The results are presented as Pearson correlation coefficient and associated *P* values (α = 0.05). Only the amount of gravel stones was analyzed as all others had low variance (pecking stones: 1 stone/1,000 hens in 100% of flocks; toys: evenly distributed in 74% of flocks; grains: 1 g/hen/day in 89.6% of flocks; oyster shells: 1 g/hen/day in 87.5% of flocks). Results for these are, therefore, reported with descriptive statistics.

The relationship between the frequency of provision of each EE type and the feather loss, flock mortality, and response to the NO test were analysed using the mixed procedure with the frequency of provision as a fixed factor and the hybrid of the flocks (Lohmann LSL or Dekalb White) as a random factor. The data fit the model assumptions, for example, normal distribution of the residuals. *Post-hoc* analysis was performed with the Tukey test (Tukey's HSD test). The frequency of provision of pecking stones, toys and gravel were not analysed due to low variance. All flocks ensured pecking stones and toys were always available to the hens and only 4 flocks (9.8%) replenished the gravel stones daily or monthly instead of weekly.

## RESULTS

Table 1 presents the descriptive statistics for the feather loss and wound scores of each body part as well as for the number of hens approaching the NO during the NO test. In general, the plumage condition varied between the assessed flocks with some flocks averaging a score of 1.7 on several body parts. The most common area for featherlessness was back/wing (mean score 0.58) and breast (mean score 0.48).

**Table 1:** Descriptive statistics of feather loss scores, wound scores, NO test results, mortality percentage and environmental enrichment parameters (age when EEs were first provided and amounts provided).

Variable	N*	Mean	Std Dev	Min	Max
FL head	45	0.36	0.38	0.00	1.38
FL back/wings	45	0.58	0.50	0.00	1.71
FL breast	45	0.48	0.47	0.00	1.70
FL tail	42	0.40	0.47	0.00	1.48
FL body average	45	0.46	0.39	0.03	1.55
FL body sum	42	1.81	1.50	0.12	6.19
Dirtiness	45	0.04	0.08	0.00	0.30
Wounds head	45	0.00	0.01	0.00	0.04
Wounds back	45	0.00	0.01	0.00	0.03
Wounds cloaca	45	0.00	0.01	0.00	0.04
Mortality	39	3.06	1.71	0.90	9.00
Hens approaching NO	45	19.26	15.80	0.25	74.25
Age EE was first given (wk)					
Pecking stone	36	24.92	3.01	16.00	35.00
Gravel	43	21.95	4.38	16.00	35.00
Toys	26	23.92	7.16	16.00	50.00
Oyster shells	38	23.50	10.22	18.00	70.00
Grains	38	26.55	4.69	16.00	35.00
Amount of EE given					
Gravel (g/hen/month)	31	3.26	2.79	1.00	10.00
Pecking stone (per 1,000 hens)	30	1.00	0.00	1.00	1.00
Oyster shells (g/hen/day)	24	1.39	1.22	1.00	6.25
Grains (g/hen/day)	29	1.10	0.76	0.50	5.00

Total number of flocks visited: 45.

Abbreviations: EE, environmental enrichment; FL, Feather loss; NO, novel object.

⁎ Number of available data points.

A positive correlation was found between the feather loss score of the tail and the age of first provision of toys (Pearson correlation coefficient = 0.41; *P* = 0.051, Table 2), with flocks having higher feather loss scores for the tail (i.e., more tail feathers missing) when toys were provided at later weeks of life. No correlation was found between feather loss scores of any body part and the age when pecking stones, gravel, oyster shells, or grains where first provided (*P* > 0.05). Wounds were rarely observed. In general, there was little variation between flocks with regards to age of first EE provision and the quantity given throughout the production period (Table 1). In addition, mean flock mortality was not found to be associated with any of the EE parameters investigated. Finally, there was no correlation between the number of hens approaching the NO and the age at which each type of EE was provided (*P* > 0.05, Table 2).

**Table 2:** Pearson correlation coefficient, *P* value, and N, the number of flocks tested.

	Age EE was first given						Amount of EE
Pearson Correlation		Pecking stone	Gravel	Toys	Oyster shells	Grains	Gravel
Head	Coeff	0.10	0.01	0.29	−0.22	0.14	−0.33
	P value	0.57	0.95	0.15	0.19	0.40	0.07
	N	36	43	26	38	38	31
Back/wings	Coeff	0.02	−0.19	0.24	−0.23	−0.18	−0.25
	P value	0.92	0.22	0.23	0.17	0.28	0.18
	N	36	43	26	38	38	31
Breast	Coeff	0.03	−0.06	0.21	−0.20	−0.03	−0.05
	*P* value	0.88	0.72	0.30	0.22	0.88	0.79
	N	36	43	26	38	38	31
Tail	Coeff	0.15	−0.13	**0.41**	−0.04	−0.22	−**0.43**
	*P* value	0.40	0.44	**0.05**	0.80	0.20	**0.02**
	N	33	40	**23**	35	35	**28**
Mortality	Coeff	−0.14	-0.21	0.34	−0.17	0.05	0.08
	*P* value	0.44	0.21	0.12	0.34	0.77	0.7
	N	32	37	22	32	33	27
NO test	Coeff	0.23	−0.11	−0.03	0.06	0.26	−0.12
	*P* value	0.17	0.50	0.90	0.73	0.11	0.52
	N	36	43	26	38	38	31

Significant correlations in bold.

Abbreviations: EE, environmental enrichment; NO, novel object.

The feathers of the tail had better condition (i.e., lower feather loss score) with increasing amounts of gravel stones (Pearson correlation coefficient = −0.43, *P* = 0.02). The frequency of provision of oyster shells and grains did not affect the mortality, the number of hens approaching in the NO test or the feather loss score of any of the assessed body parts (*P* > 0.05, Table 3).

**Table 3:** Results (LS means ± SE, F ratios, and *P* values) of the effects of the frequency of provision of oyster shells and grains spread on the litter on the feather loss (FL) scores for the different body parts, mortality, and on the number of hens approaching the novel object during the NO test.

	Daily		Weekly		Monthly		Statistics	
	LS mean	SE	LS mean	SE	LS mean	SE	F ratio	*P* value
FL head								
Oyster shells	0.27	0.18	0.41	0.17	−0.03	0.29	2.06	0.14
Grains	0.34	0.19	0.26	0.21	0.38	0.25	0.19	0.83
FL back/wings								
Oyster shells	0.67	0.19	0.50	0.18	0.28	0.38	0.79	0.46
Grains	0.43	0.27	0.44	0.30	0.61	0.33	0.24	0.79
FL breast								
Oyster shells	0.42	0.24	0.44	0.23	−0.15	0.37	1.87	0.17
Grains	0.35	0.28	0.31	0.30	0.48	0.32	0.22	0.81
FL tail								
Oyster shells	0.47	0.20	0.27	0.19	0.63	0.35	1.20	0.32
Grains	0.30	0.20	0.38	0.21	0.17	0.32	0.24	0.79
Mortality								
Oyster shells	2.7	0.5	3.3	0.5	1.44	1.7	0.91	0.41
Grains	3.0	0.7	2.9	0.95	2.12	1.0	0.35	0.71
NO test								
Oyster shells	23.13	7.22	18.01	6.94	39.76	13.34	1.80	0.18
Grains	25.08	9.62	23.00	10.47	22.62	11.71	0.07	0.94

Abbreviations: FL, Feather loss; NO, novel object.

## DISCUSSION

The aim of the study was to investigate the associations between commercially applied environment enrichment and selected measures of behavior and welfare in flocks of loose-housed laying hens. We expected that increased environmental complexity (i.e., earlier age, increased quantity, and frequency of EE provision) would result in improved plumage condition and reduced fearfulness. The results showed that providing toys, such as cut-up pieces of old manure belt, cardboard pieces, and plastic balls in various colors, at an early age (e.g., 16–20 wk of age) was correlated with a reduction in the amount of tail damage at the end of the production period (70–76 wk of age). It is well established in the literature that early life experience is a key factor in the behavioral development of the laying hen. Early life exposure to environmental complexity and resources has been shown to affect behaviors such as perching (Gunnarsson et al., 2000), cloacal cannibalism (Gunnarsson et al., 1999), feather pecking (de Haas et al., 2014; Tahamtani et al., 2016), fearfulness (Anderson and Adams, 1994; de Haas et al., 2014; Brantsæter et al., 2017), and laying eggs on the floor (Gunnarsson et al., 1999). The present study, therefore, adds to this knowledge suggesting that the use of EE is most beneficial against tail damage when provision starts earlier rather than later.

The results also showed a positive effect of the amount of gravel stones provided to the flocks on the condition of the tail feathers. Gravel stones, or grit, are positive for chicken digestion. Provision of grit can improve growth, feed efficiency, and egg production (Balloun and Phillips, 1956). However, there is no evidence that access to grit directly affects the incidence of feather pecking behavior. The results seen in the present study are most likely due to gravel provision functioning as an added pecking substrate onto which the hens focus their foraging and pecking activity, thereby reducing the frequency of feather pecking. As previously mentioned, feather pecking develops as a result of thwarted motivation for either foraging or dustbathing, resulting in a redirection of pecks from the litter to the feathers of conspecifics (Blokhuis, 1986; Vestergaard and Lisborg, 1993). Thus, the performance of this behavior indicates reduced welfare of the feather pecker as well as the feather pecked. Therefore, enrichment objects which supplement the quality and complexity of the litter can have valuable effects in minimising this behavior and improving welfare in the whole flock.

Obtaining a reliable measurement of the level of severe feather pecking behavior in large commercial flocks is time-consuming, as performance of the behavior may be sporadic and vary with time of day (Kjaer, 2000). However, assessing the damage to the plumage of laying hens caused by severe feather pecking has long been considered a valid and useful indicator of the level of severe feather pecking behavior in the flock. For example, Bilcik and Keeling (1999) found a positive association between the number of severe feather pecks received and the degree of plumage damage. Several studies have shown that plumage damage increases with age (Nicol et al., 2006; Drake et al., 2010; Hinrichsen et al., 2016). This may be due to wear of the plumage, accumulation of plumage damage or an escalation of severe feather pecking behavior, but the majority of the damage has been confirmed to be due to feather pecking (Bilcik and Keeling, 1999). Damaging feather pecking is typically directed mainly at the back and vent area while feather damage to the head and neck can be due to abrasion or aggression around limited resources (Bilcik and Keeling, 1999; Heerkens et al., 2015). In our study, the back and breast areas had worse plumage score while the head was the area least affected. This indicates that aggression and aggressive pecking was not responsible for the bulk of plumage damage observed in this study. Instead, the results from this study indicate that improving the foraging opportunities with the use of EE can best address this issue.

The lack of associations between the provision of EE and plumage score of the head, back/wings, and breast areas is likely due to the lack of variation in the provision of EE. As EE provision now is part of the laying hen welfare scheme for most companies in Norway, all visited production farmers generally provided the same type and quantity of EE, making it difficult to detect differences. It would be important, therefore, to perform similar surveys in farms or countries that have a more varied attitude to the use of EE. Nevertheless, despite the wide use of a variety of EE during the production period, plumage damage due to feather pecking was still observed, with some flocks having as high average plumage damage scores as 1.71 of 2. One important thing to note is that no information is known regarding the access to EE the flocks assessed here had during the rearing period (i.e., 0–16 wk of age). As previously mentioned, early life experience with EE and complexity has significant and long-lasting effect on the behavior of laying hens. Considering, therefore, that the use of EE is already quite extensive during the production period, the provision of EE during the rearing period is likely to be an effective method of further reducing the incidence of feather pecking.

Despite the large body of previous literature showing that environmental complexity and EE reduce fearfulness, we did not find any effect of the age point, quantity, or frequency of EE provision on the results from the novel object test. There was a relatively large variation in the number of hens approaching the NO, but little variation in the provision of EE in the production farms. This suggests that other factors influence the development of fearfulness, for example the environment that the hens experience during the rearing period (de Haas et al., 2014; Brantsæter et al., 2017). High fearfulness of the flock can have serious welfare and economic consequences. For example, increased fearfulness has been associated with severe feather pecking (Vestergaard et al., 1993; Jones et al., 2005). Also, high fearfulness can lead to panic-induced smothering and avian hysteria (also known as social clumping) which has been described as unexplained extreme nervousness followed by squawking, flight and the hiding or crowding in corners and under feeders (Sanger and Hamdy, 1962; Bright and Johnson, 2011). One study of UK free-range farms reported smothering events in approximately 60% of flocks and an average loss of 25.5 birds per incidence (Barrett et al., 2014). Furthermore, smothering is most often reported to occur on the litter and in corners of the house (Richards et al., 2012; Barrett et al., 2014) and may, therefore, occur in all production systems. Consequently, there is a growing interest in the Norwegian egg sector to understand social clumping and find measures to avoid this behavior from developing in the flock.

Another relevant development of the egg sector is the growing interest in extending the production period of laying hens from the current 78 wk to 90–100 wk (Bain et al., 2016). This strategy is seen as an efficient way to address the growing demand for food production and to become a more sustainable production. It is estimated that this extension of the laying period could reduce the size of the UK flocks, including the parent stock, by 2.5 million birds per year (Bain et al., 2016). However, such an extension of the laying period incurs obstacles such as poor egg quality, osteoporosis, keel bone integrity, feather pecking, and mortality rate (Alfonso-Carrillo et al., 2021; Wall et al., 2021). It is, therefore, vital that the welfare of commercially housed laying hens be assessed at the current end of lay (e.g., 70–78 wk) in order to determine the challenges that must be overcome in order to extend the production period of these birds without further compromising their welfare.

In conclusion, the present study showed that the provision of EE objects such as toys and gravel stones can have significant benefits to the condition of laying hen plumage. In particular, the positive effects of these enrichment objects increases if they are provided in early life and in larger quantities. This study also adds to the body of literature supporting the importance of early life experiences on the behavioral development of laying hens and on their welfare at older ages.
